# Modeling versatility as the hallmark of model organisms

**DOI:** 10.1007/s40656-026-00718-5

**Published:** 2026-02-24

**Authors:** Guido I. Prieto, Alejandro Fábregas-Tejeda

**Affiliations:** 1 Department of Philosophy, Bielefeld University, Postfach 100131, 33501 Bielefeld, Germany; 2 Centre for Logic and Philosophy of Science, Institute of Philosophy, KU Leuven, Kardinaal Mercierplein 2 - box 3200, 3000 Leuven, Belgium

**Keywords:** Model organism, Modeling, Model carrier, Scientific representation, Representational power, Representational scope, Ontogeny

## Abstract

In recent years, discussions on the epistemology of model organism-based research have emerged in the philosophy of science. A key topic of discussion is how the epistemic insights gained from model organisms differ from those gained through other experimental organisms used in laboratory and field research. Here, we argue that model organisms are epistemically special due to their nature as ontogenetically changeable, standardized, and evolved material *model carriers*. These characteristics afford six important kinds of *modeling versatility* that biologists marshal in their investigations: (i) synchronic target versatility; (ii) synchronic scope versatility; (iii) diachronic target versatility; (iv) diachronic scope versatility; (v) manipulation versatility; and (vi) discovery versatility. In presenting these dimensions of modeling versatility, we also clarify key notions such as ‘representational target,’ ‘representational scope,’ and ‘representational power’ as these apply to modeling practices that involve model organisms.

## Introduction

Prevailing empirical practices within the biological and biomedical sciences could not be understood without the use of a swathe of so-called *model organisms*, such as the fruit fly *Drosophila melanogaster*, the African clawed frog *Xenopus laevis*, and the thale cress *Arabidopsis thaliana*. Recognizing its undeniable heuristic and explanatory importance, both scientists and philosophers of science have paid attention to different facets of model organism-based research (for comprehensive overviews, see Ankeny & Leonelli, 2020; Green, 2024).

An important topic of discussion is how the epistemic insights gained from model organisms differ from those gained from other experimental organisms employed in empirical investigations in the laboratory and the field.^1^ For instance, Rachel A. Ankeny and Sabina Leonelli (2011, 2020) proposed that what distinguishes model organisms from other experimental organisms is their relatively higher *representational power*, which stems from the combination of broad *representational target* (i.e., the number of phenomena that a model organism can represent) and broad *representational scope* (i.e., the extent to which the result of studies on a model organism can be extrapolated to other organisms). Its value notwithstanding, Ankeny and Leonelli’s account rests on the assumption that model organisms are *bona fide models*, which is a contested issue in philosophical scholarship (for arguments for and against this idea, see, among others, Levy & Currie, 2015; Parkkinen, 2017; Sartori, 2023; Prieto & Fábregas-Tejeda, 2025).

We argued elsewhere (Prieto & Fábregas-Tejeda, 2025) that, strictly speaking, model organisms are *not* models, but they nonetheless play a crucial representational role by supporting the construction of manifold models that represent target phenomena in other organisms. We formalized this idea by refining and then implementing the DEKI account of scientific representation.^2^ Specifically, we conceptualized model organisms as model *carriers*, some parts of which are abstracted and picked out for experimental study. These carrier parts embody the phenomena of interest for researchers and serve as the basis for the construction of models by the interpretation of these parts as causal processes or mechanisms within certain theoretical domains—i.e., a model is defined as *M =* (*X*^*I*^, *I*), whereby *X*^*I*^ is the interpreted *part* of a carrier *X* and *I* is the interpretation of *X*^*I*^ in terms of a certain domain *Z*. The resulting models can be used to represent the target phenomena—which presumably bear some similarity to the phenomena investigated in the model organism (e.g., they are homologous, analogous, structurally similar, or functionally equivalent)—in other organisms (see also Kunze & Malfatti, 2025).

**Fig. 1:**
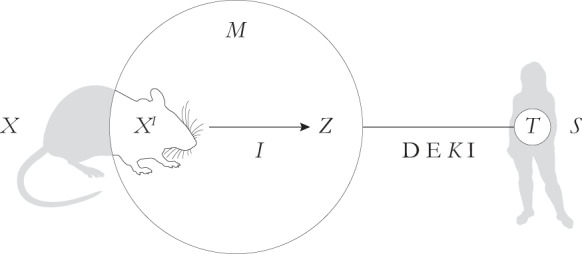
Representing with model organisms. In model organism research, a model organism plays the role of a model carrier (*X*), only a selected and abstracted part of which (*X*^*I*^) is interpreted (*I*) in terms of a given theoretical domain (*Z*). The resulting model (*M*) can then be used to represent a target phenomenon (*T*) in another organism (*S*) that is known or assumed to be in some way similar to the model organism’s part. Although in this paper we can safely ignore the details of how the relation between model and target is enacted, it is worth mentioning that, according to the DEKI account of representation, a model represents a target if it denotes it (D) and exemplifies (E) a set of features that are usually transformed via a key (*K*) before being imputed (I) to the target. For a thorough analysis, see Prieto and Fábregas-Tejeda (2025)

In short, under this view, representation with model organisms involves the construction of models from selected parts of model organisms to represent circumscribed target phenomena in other organisms (Fig. 1). This idea contrasts with previous accounts (e.g., Ankeny & Leonelli, 2020; Sartori, 2023) that portray model organisms either as models or as carriers wholly included in models—i.e., *M =* (*X*, *I*), whereby *X* is a *whole* carrier and *I* is its interpretation in terms of a certain domain *Z*—and that construe targets as whole organisms (see Prieto & Fábregas-Tejeda, 2025, for in-depth discussion).

In this article, we mobilize our refined version of the DEKI account of model organisms as carriers (Prieto & Fábregas-Tejeda, 2025) to shed light on the differences between model organisms and other experimental organisms.^3^ Our central claim is that the distinctive feature of model organisms *qua* carriers should not be sought in their ‘representational power’ (*sensu* Ankeny & Leonelli, 2011, 2020)—model organisms cannot be attributed representational power, we contend, because they are not models in the first place—but in their *modeling versatility*. This characteristic is primarily grounded on the not-so-trivial fact that model organisms are *standardized organisms*—i.e., material, integrated systems with an evolutionary history and an organization that changes over the course of their ontogeny, which also exhibit some traits that are generated through standardization and preparatory experimentation.

We start by presenting, discussing, and clarifying Ankeny and Leonelli’s notions of ‘representational target’ and ‘representational scope’ (Sect. 2). Then, we use our modified versions of these concepts—which we call ‘*T*-scope’ and ‘*S*-scope,’ respectively—to introduce four types of modeling versatility that model organisms possess. These are: ‘synchronic target versatility’ and ‘synchronic scope versatility’ (Sect. 3), and their diachronic equivalents, ‘diachronic target versatility’ and ‘diachronic scope versatility’ (Sect. 4). Next, we move on to expound the process of standardization of model organisms in the laboratory as modification of the pool of carrier-features (Sect. 5), which is necessary for introducing two further types of versatility: ‘manipulation versatility’ and ‘discovery versatility’ (Sect. 6). We conclude with a discussion on modeling versatility *vis-à-vis* representational power and the differences between model organisms and other types of experimental organisms (Sect. 7).

## Two types of scope

A primary aim of model organism-based research is to conduct thorough cross-species work by using and integrating diverse disciplinary approaches and methods. This involves a strategic accumulation of resources and infrastructure on particular organisms (e.g., the African clawed frog *Xenopus laevis*, the roundworm *Caenorhabditis elegans*, the spreading earthmoss *Physcomitrella patens*, and so on) that are repeatedly studied. Subsequently, a given model organism serves as a key reference point for launching comparisons between, and drawing inferences about, organisms from different species. In this sense, model organisms are employed in biological science with the goal of generating knowledge that is valid beyond the focal organisms under study in concrete practices (for discussion, see Kunze & Malfatti, 2025).

Ankeny and Leonelli (2011, 2020) contend that this epistemic extension occurs concurrently along two dimensions they call ‘representational target’ and ‘representational scope.’ By *representational target*, they refer to “the phenomena to be explored through the use of the experimental organism” (Ankeny & Leonelli, 2011, p. 315), that is, the array of phenomena earmarked by scientists as being worthy of investigation. In contrast, the *representational scope* is a form of inductive-inference generation that is partially reliant on evolutionary conservation and captures “how extensively the results of research with a particular experimental organism (a specimen or token) can be projected onto a wider group of organisms (a type)” (Ankeny & Leonelli, 2011, p. 315). For example, under this view, the phenomenon of limb regeneration is part of the representational target of the axolotl *Ambystoma mexicanum qua* model organism, whereas its representational scope extends as far as the models of limb regeneration apply across the Urodela, and perhaps to other tetrapods, including humans (Roy & Gatien, 2008; Zhong et al., 2023). Ankeny and Leonelli (2011, p. 315) summarize the difference between the representational target and representational scope by indicating that “while the representational target describes the conceptual reasons why researchers are studying a given organism, the representational scope defines the extent to which researchers see their findings as applicable across organisms.”

While insightful, the notions of representational target and representational scope would benefit from further clarification. In particular, we find two sources of ambiguity surrounding the idea of representational target:^4^ (i) Ankeny and Leonelli do not always distinguish it from the notion of a target *tout court*; and (ii) there are several passages in their writings where both the representational target and the target are identified either as an “organism taken as a whole” (Ankeny & Leonelli, 2020, p. 26) or as a circumscribed phenomenon, such as when they say that “anything from ‘metabolism’ to a ‘[*Hox*] gene’ constitutes a phenomenon and can become the representational target” (p. 8).

Instead, we want to underscore that (i) the notions of representational target and target are distinct, and (ii) neither refers to whole organisms. On the one hand, a target is a *phenomenon* that occurs in an organism or—with some variation—in a collection of organisms (rather than a whole organism) that is assumed to be somewhat similar or functionally analogous to the phenomenon investigated in a model organism (see Prieto & Fábregas-Tejeda, 2025; for discussion on the importance and meaning of similarity for model organism-based research, see also Kunze & Malfatti, 2025). On the other hand, a representational target, in the framing offered by Ankeny and Leonelli, is the *set of targets* that can be represented through models constructed using a given model organism. Thus, the representational target could be thought of as the “target scope” or “model scope” of a given model organism. In other words, this notion should capture the idea: ‘one model organism (*qua* carrier), many models (or, equivalently, many targets).’

To avoid confusion with the notion of target, and to capture its scope character, we rename and clarify the concept of representational target as:

***T*-scope:** A *T*-scope is the set of target phenomena *T*_*i*_ that are amenable to being represented by models *M*_*i*_ *=* (*X*^*I*^_*i*_, *I*_*i*_) as *Z*_*i*_ using abstracted parts *X*^*I*^_*i*_ of a given carrier *X*.

The *T*-scope can be predicated on the models constructed from a model organism at a specific time or ontogenetic stage. Occasionally, it might also be useful to talk about the *total* T*-scope* as the union of the *T*-scopes of all the models built from a model organism throughout its life cycle (see Sect. 4.1).

The notion of *T*-scope follows straightforwardly from our account of model organisms as partially interpreted carriers (Prieto & Fábregas-Tejeda, 2025). Indeed, this account highlights the possibility that several target phenomena are represented using a given model organism through the construction of models based on the abstraction and selection of non- or partially-overlapping *parts* (*X*^*I*^_*i*_) of the same model organism (*X*). The process of abstraction and selection of different carrier-parts is guided by the domains *Z*_*i*_ that capture the representational motifs of the models, which thus play the role of what Rasmus G. Winther (2006) calls ‘partitioning frames,’ i.e., “set[s] of theoretical and experimental commitments to […] particular way[s] of abstracting kinds of parts” (Winther, 2011, p. 401, emphasis removed).

Concerning the notion of ‘representational scope,’ the problem with Ankeny and Leonelli’s definition is that it is predicated on a model organism. Instead, we think that the representational scope should not be interpreted as a property of model organisms per se, but of *each of the models* that are constructed using a given model organism to represent different targets. Put simply, the notion of representational scope should encapsulate the idea: ‘one model (or, equivalently, one target), many organisms.’ To be clear, we propose to reinterpret the representational scope as:

***S*-scope:** An *S*-scope is the set of systems (e.g., organisms) or types of systems (e.g., taxa) *S*_*j*_ in which instances of a given target *T* represented by a model *M =* (*X*^*I*^, *I*) are known to occur or presumed to occur.

**Fig. 2:**
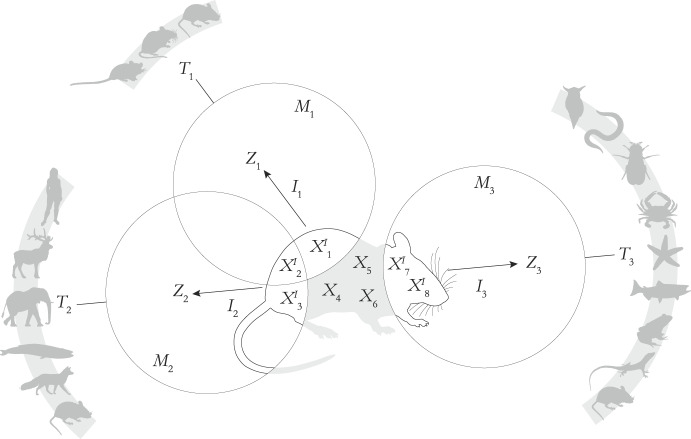
Synchronic target and scope versatilities. The diagram shows a hypothetical case of a model organism serving as the carrier of three models *M*_1_ = ({*X*_1_^*I*^, *X*_2_^*I*^}, *I*_1_), *M*_2_ = ({*X*_2_^*I*^, *X*_3_^*I*^}, *I*_2_), and *M*_3_ = ({*X*_7_^*I*^, *X*_8_^*I*^}, *I*_3_), each consisting of an interpretation of some carrier-features within a certain theoretical domain *Z*_1_, *Z*_2_, or *Z*_3_, respectively. The models represent target phenomena *T*_1_, *T*_2_, and *T*_3_ that occur in different sets of organisms (in gray). In this example, the *T*-scope is the set of targets that are represented using the model organism, i.e., *T*-scope = {*T*_1_, *T*_2_, *T*_3_}. The *S*-scope of each model is the set of organisms in which the respective target phenomenon is known or assumed to occur. In the example, the *S*-scope of *M*_1_ is, roughly, ‘murids,’ *M*_2_’s is ‘mammals,’ and *M*_3_’s is ‘metazoans.’ We can also evaluate the time-indexed *S*-scope as the union of the *S*-scopes of all the models. In the example, since the set ‘murids’ is included in ‘mammals’ and this in ‘metazoans,’ the time-indexed *S*-scope would be ‘metazoans.’ Notice that models can partially overlap and share *X*-features (e.g., both *M*_1_ and *M*_2_ include feature *X*_2_^*I*^). Also, it is worth noting that a part of a carrier might not be covered by any model (e.g., the set of non-interpreted features {*X*_4_, *X*_5_, *X*_6_}). In real case scenarios of scientific modeling, the number of interpreted and non-interpreted carrier-features is generally higher than the one shown here as an illustration. Lastly, the DEKI account of scientific representation imposes no restriction on what counts as ‘features,’ so they should not be understood as isolated, discrete items (see Prieto & Fábregas-Tejeda, 2025)

Although the *S*-scope is predicated of each of the models built from model organisms, one could nevertheless want to refer to a *time-indexed* S*-scope*—the union of the *S*-scopes of all the models at a specific time or developmental stage—or even to a *total* S*-scope*—the union of all the models generated from a model organism throughout its entire ontogeny (see Sect. 4.2). However, we think that these notions are usually less useful than the model-specific *S*-scope defined above because they mask the fact that different models constructed with a certain model organism may have radically different scope and applicability across the tree of life (see Fig. 2).

Let us now use the concepts of *T*-scope and *S*-scope to spell out four dimensions of modeling versatility that characterize modeling practices which employ model organisms, first by presenting a synchronic, static view (Sect. 3) and then by outlining a diachronic, dynamic view (Sect. 4).

## The synchronic picture of modeling versatility

At a given stage in its life cycle, a model organism can play the role of carrier of manifold models that represent diverse target phenomena—i.e., it can have a broad *T*-scope. Moreover, each of the models can have a variable *S*-scope, thus capturing specific phenomena that are presumed to occur in diverse groups of organisms (Fig. 2). We call these dimensions of modeling versatility of model organism-based research *synchronic target versatility* and *synchronic scope versatility*, respectively. Let us tackle each of them sequentially.

### Synchronic target versatility

James R. Griesemer (1990) has argued that “material models are able to serve certain sorts of theoretical functions *more* easily than abstract formal ones in virtue of their material link to the phenomena under scientific investigation,” especially because the features of the carriers in question, say, model organisms, are “literally embodiments of [the target] phenomena” (p. 80), or at least good approximations of them in spite of certain degrees of evolutionary divergence (e.g., between *Mus musculus* and *Homo sapiens*). In the context of model organism-based research, this means that at a particular moment in its ontogeny, a model organism *qua* carrier instantiates several processes and possesses many different features that can be used to study equally diverse target phenomena that constitute its *T*-scope. We call this first dimension of the modeling versatility afforded by model organisms *synchronic target versatility*.

The idea that an organism at a given developmental stage can support different models aimed at representing different targets—i.e., that it can have synchronic target versatility—departs definitively from the literal interpretation of model organisms as models (as criticized in Prieto & Fábregas-Tejeda, 2025).^5^ In actual scientific practice, a single model organism can be, and customarily is, used as the carrier of many different models. For instance, adult mice (*M. musculus*) have been used to study, among countless target phenomena, metabolic and monogenic diseases, histocompatibility, immunological defenses to pathogens, different sorts of behaviors, and cancer development (Guénet, 2005; Merlo et al., 2012). Similarly, adult crickets are used to build projectable models for, among other phenomena, leg regeneration, hormonal circadian rhythms, nervous system plasticity, and social aggression behaviors (see Horch et al., 2017, and chapters therein).

These considerations challenge not only the literal interpretation of model organisms as models but also previous interpretations of DEKI by Ankeny and Leonelli (2020) and Lorenzo Sartori (2023), in which the whole carrier organism is taken to represent other whole organisms as targets (for a detailed articulation and critique of this view, see Prieto & Fábregas-Tejeda, 2025). On this point, we want to emphasize that being able to build a constellation of models of different targets in a certain organism is simply not synonymous with representing that organism as a “whole.” A *token* lab mouse will never represent a “whole *H. sapiens*:” only certain models built in, within, and through *M. musculus* represent certain parts and processes of human physiology, development, pathology, etc. In short, model organisms always yield fragmentary, target-specific models of other organisms—their incomplete and directed character is, after all, a shared characteristic of all scientific models (for further discussion, see Prieto & Fábregas-Tejeda, 2025).

Unlike the cases of the literal interpretation of model organisms as models and previous accounts using DEKI, synchronic target flexibility follows naturally from our modified version of DEKI (Fig. 2; Prieto & Fábregas-Tejeda, 2025). Specifically, we have contended that only the interpreted part of a carrier is *de facto* included in a model (together with its interpretation), and that the model represents a part of (i.e., target in) other organisms. This allows for *different parts* of the carrier (i.e., different sets of *X*-features) to be interpreted separately in terms of different *Z* domains, which effectively results in different models aimed at different targets in one or more organisms in the *S*-scope. Importantly, the sheer complexity of organisms and our epistemic limitations to grasp it—which can be done only partially by foregrounding specific phenomena and abstracting away other phenomena within certain partitioning frames—guarantee that there will always be non-interpreted parts of the carrier (i.e., currently not covered by scientific models) that might be used as bases for new models at a future time. In this sense, organisms *qua* carriers are open-ended (more on this in Sect. 6).

It should be brought to mind that the different models sharing a given carrier need not be entirely disjoint. In fact, they usually share some *X*-features—albeit sometimes interpreted differently across various domains—and provide each other with theoretical and empirical background conditions. Different models can also target the same phenomenon, provided they interpret the same part of the carrier differently (i.e., they differ in *I* and *Z*) or that they include non-identical parts of the carrier (i.e., they differ in the set of *X*-features they include and therefore in *I*; e.g., when a more detailed model includes some extra features that simpler models of the same target exclude). The accumulation and coexistence of different models on the same carrier organism creates the conditions for establishing points of contact between the models to arise and build up, which grounds the role of model organisms as “integrative platforms” (Ankeny & Leonelli, 2020, p. 24).

### Synchronic scope versatility

There is another dimension of modeling versatility of model organisms that follows from our account (Prieto & Fábregas-Tejeda, 2025). According to this vantage point, in model-organism based research, a model represents a target system that is not a whole organism but a phenomenon in an organism. Thus, the target of a model need not be restricted to organisms of a single species; rather, the same phenomenon (or sufficiently similar or conserved phenomena) that is the target of the model can be found in different, even phylogenetically distant species. In other words, when a model is used as a representation-of a target, it has a certain *S*-scope determined by the extent to which its target is present in other species or taxa. As we saw above, different models can be built on a single carrier organism, each with a specific *S*-scope. This means that, at a particular moment in its ontogenetic trajectory, a carrier organism can support several models, each with varying *S*-scope (Fig. 2). We call this the *synchronic scope versatility* afforded by model organisms as carriers.^6^

Far from being a mere conceptual corollary of our account, synchronic scope versatility is a pervasive dimension in model organism research. For instance, the plethora of models derived from *M. musculus*-based research differ ostensibly in their *S*-scope. Some have a quite narrow *S*-scope: for example, models restricted to murid rodents (e.g., Wang et al., 2020), or mouse models of diseases that aim to have a *S*-scope that basically encompasses only one species in its adult form, namely *H. sapiens* (e.g., Rydell-Törmänen & Johnson, 2019). In contrast, when scientists study, say, sensory processing mediated by vibrissae in adult *M. musculus*, the intended *S*-scope of their models include all mammals *except* the whiskerless monotremes (duck-billed platypus and echidnas) and humans (see Diamond et al., 2008). Finally, there are models built with this same carrier organism with yet a broader *S*-scope: for instance, the models of the so-called “prefrontal operations” in the adult mouse pre-frontal cortex are taken to hold across mammals despite cytoarchitectonic differences (Le Merre et al., 2021).

Of course, this is not to say that models necessarily have different *S*-scope: there can be models with the same *S*-scope, either because they share the same target or because they target different phenomena that happen to be manifested in the same group of organisms. The important point here is that carrier organisms are *versatile* in the sense that they can, in principle, support models with varying *S*-scope.

Moving forward, we need to appreciate another important intrinsic element of model organisms as carriers so that additional dimensions of modeling versatility can come into full view: model organisms are developing systems.

## The diachronic picture of modeling versatility

**Fig. 3:**
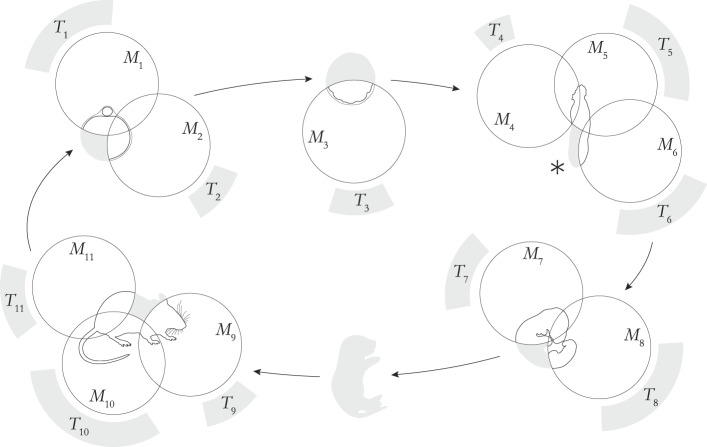
Diachronic target and scope versatilities. The diagram shows a hypothetical case of six stages in the life cycle of a model organism, each depicted as a simplified version of the diagram in Fig. 2. At different stages, the organism functions as a carrier of models that represent target phenomena in other organisms. For example, at the developmental stage marked with (*), the *T*-scope is the set of target phenomena {*T*_4_, *T*_5_, *T*_6_} represented by the models *M*_4_, *M*_5_, and *M*_6_, whereas the *S*-scope of each model is the set of organisms in which the corresponding targets occur (depicted as gray ranges of varying length). We can also characterize a time-indexed *S*-scope as the union of these *S*-scopes. Taking the entirety of the model organism’s ontogeny, the total *T*-scope is the set of targets {*T*_1_, …, *T*_11_} and the total *S*-scope is the union of the *S*-scopes of models *M*_1_, …, *M*_11_. The toy figure also shows that, in principle, there can be parts of the organism or even entire developmental stages not covered by any available scientific model

The fact that model organisms are developing systems means that their physiology, morphology, behavior, ecological interactions, and cognition undergo changes throughout their ontogenies. In the context of the representational practices of the life sciences in which model organisms partake, this entails that the set of *X*-features that a model organism has at any given time changes as a function of development, thus opening up the horizon of possible representations and what is (or is not) *de facto* interpreted within scientific models. The changing sets of *X*-features that can be theoretically interpreted in different domains and mobilized into manifold models have consequences, we argue, for both the *T*-scope and the *S*-scope (Fig. 3). Let’s explore them sequentially.

### Diachronic target versatility

The dimension of what we call *diachronic target versatility* can be explained by highlighting that the *X*-properties of organisms radically change throughout their ontogeny, and in each developmental stage, biologists can assemble different models for varied target phenomena. In other words, the *T*-scope of a model organism varies with its developmental stage.

If we take the example of *M. musculus*-based research (Fig. 3), not all possible targets are exhausted by the models that can be derived through adult stages of the mouse. Many important target phenomena are restricted to particular developmental timeframes in the ontogeny of *M. musculus* (e.g., fertilization, segmentation, gastrulation, and organogenesis) and thus can only be boundedly studied when the model carrier possesses certain features.^7^ For example, the famous ‘clock and wavefront’ model provides a working hypothesis of how the target phenomenon of ‘somitogenesis,’ i.e., the rhythmic segmentation of mesoderm into somites,^8^ comes into being through the action of transcriptionally oscillating genes in the axis of vertebrate embryos. An adult *M. musculus* cannot act as a carrier for models of somitogenesis: it simply does not possess the features that can be theoretically interpreted for capturing and understanding somite formation, nor exemplifies any relevant features thereof. The process of somitogenesis can only be considered with proper diachronic contextualization of theoretically interpreted, exemplified, and keyed-up features in mouse embryos that can be imputed to vertebrate embryos more generally (see, e.g., Ibarra-Soria et al., 2023). Likewise, although it sounds obvious, the target ‘adult neurogenesis,’ investigated for its potential links to learning, memory, and neurodegenerative disorders in humans, can only be modeled by taking into account the structures and processes within the brains of *adult* mice.

This diachronic versatility of available targets of model organisms at different developmental moments extends to behavior as well: when employing *M. musculus*, the study and modeling of different behaviors (and their variability, causal underpinnings, and environmental sensitivity) can only be achieved in particular windows of its ontogeny (for an overview, see Brust et al., 2015). In sum, model organisms have varying ontogenetic features that open different lines of inquiry, and models therefrom, for scientists.

### Diachronic scope versatility

The changes that model organisms undergo throughout their ontogenies also have an impact on the *S*-scope of the models that can be built by theoretically interpreting selected parts of the model organism at different stages in its life cycle. This is what we refer to as the *diachronic scope versatility* dimension (Fig. 3).

Coming back to our example, the variable characteristics that are generated throughout the ontogeny of *M. musculus*, as is the case with any model organism, also influence the *S*-scope of the models that can be constructed with this material carrier. For instance, although the mouse gastrula is taken to be somewhat unique in its morphology, the morphogenetic models of gastrulation (e.g., of cellular interactions, cell migration, and physico-mechanical and genetic-molecular mechanisms yielding germ layer formation) are deemed to represent gastrulation in all mammals (for discussion, see Tam & Behringer, 1997) but not in all metazoans. For understanding the phylogenetic diversity of animal gastrulation, the insights from several model carrier organisms and non-model species need to be integrated to arrive at large-scale models (see Serrano Nájera & Weijer, 2023). In contrast to mouse gastrulation, other developmental models have a wider *S*-scope: for example, the left-right symmetry breaking model whereby directional fluid flow of secreted factors generated by motile cilia at the left-right organizer (the so-called “the node”) converts cellular chirality to embryo-level asymmetry (Nakamura et al., 2006, 2012) is assumed to be at play for many important groups of vertebrates (see Hamada & Tam, 2020). And we could also cite another example with an even broader *S*-scope: the models of notochord signaling—a transient embryonic structure whose morphogenesis starts mid-gastrula in *M. musculus*—related to midline patterning and the dorsal-ventral patterning of the neural tube purportedly extend to all members of the phylum Chordata (Balmer et al., 2016; Stemple, 2005).

Importantly, besides the types of local and broad keys discussed in detail by Sartori (2023) in his exposition of the DEKI account, the imputation of model features to targets in model organism-based research strongly depends on phylogenetic considerations (e.g., homology assessments) and especially on assumptions of evolutionary conservation (for discussion on these issues, see Levy & Currie, 2015). For instance, the notochord signaling model that takes features of *M. musculus* as its carrier could hardly have a chordate-wide *S*-scope if it weren’t for the evolutionary conservation of the signaling functions of this transient embryonic structure in chordate evolutionary history. The *S*-scope of the models derived from research on model organisms cannot be untied from the evolutionary history of the *X*-features that are marshaled and interpreted in modeling endeavors in the first place.

In what follows, we shall discuss yet two additional dimensions of modeling versatility. Since these hinge upon different kinds of *X*-features that model organisms have as products of an evolutionary history and standardization processes that yield great experimental manipulability, we will devote the next section to illustrating them.

## Standardization and carrier features

To flesh out the last two dimensions of the epistemic versatility of modeling derived from model organism-based research, we need to briefly discuss how model organisms come into being in scientific practice. After all, as Robert E. Kohler (1991) has underscored, model organisms in the life and biomedical sciences are deeply tied with particular “systems of production.”

**Fig. 4:**
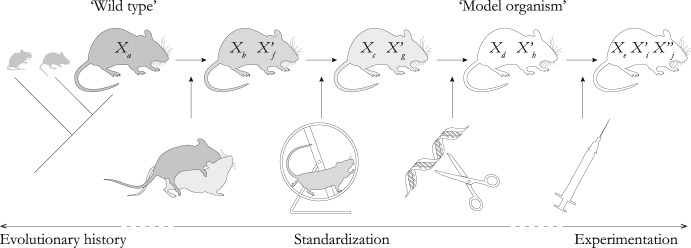
Standardization as modification of carrier-features in model organisms. The figure depicts the changes of an organism’s features as it undergoes processes of evolution, standardization, and experimentation. At different times, the organism possesses varying sets of evolved features (*X*) that are the result of its evolutionary history, standardized features (*X’*) that result from the process of standardization in the laboratory, and experimental features (*X”*) that are incorporated into the standardized model organism in particular experimental settings. These features are then marshaled by scientists to build different models (as depicted in Figs. 2 and 3) with varying (synchronic or diachronic) target and scope versatilities

Once they have been selected and brought from the wild into the laboratory by waging different sets of criteria (e.g., ease of supply, rearing costs, length of life cycle, availability of methods and techniques, ethical considerations, and translational promise; for discussion, see Dietrich et al., 2020; Kunze & Malfatti, 2025), the chosen organisms must undergo a process of standardization (especially genetic standardization) and modification to actually become ‘model organisms’ (Fig. 4). These include regimenting and simplifying their environmental settings (e.g., establishing standardized growth conditions, homogenizing diets), controlling their reproduction, sequencing and annotating their genomes, profiling their gene expression patterns in varied developmental timeframes, adapting methods to make transgenic mutants (e.g., for performing gene knock-outs) and allowing for diverse experimental manipulations (e.g., developing effectors of interest for labeling and imaging), among others (for an overview, see Matthews & Vosshall, 2020; see also Ankeny & Leonelli, 2020). It is in this phase that so-called “preparatory experimentation,” not directly aimed at hypothesis testing nor theory construction, but channeled for setting the stage for model organism-based research, unfolds (Weber, 2004, p. 174).

These targeted changes to organisms and additional standards of care and maintenance are important for long-term planning and diverse scientific uses, ease of control and manipulation, shareability of data, and to meet many representational demands of future inquiries (Ankeny & Leonelli, 2020; for a detailed account of the case of *Arabidopsis thaliana*, see Leonelli, 2007). With the passing of time, then, “an intricate array of experimental tools and methods are developed” for the model organism in question (Levy & Currie, 2015, p. 333).

Model organisms are thus embedded in rich research contexts with many material and infrastructure-linked resources at hand that contribute to the entrenchment of particular species within international research communities (Levy & Currie, 2015, p. 341). Ankeny and Leonelli (2020, p. 24) refer to these as “placelessness modeling ecosystems,” distributed in different research institutions across the world, in which models, laboratory conditions, methods, tools, and infrastructures tightly come together. These can only be built in and dispersed to different countries by hosting pertinent conferences, establishing stock centers, robust networks of exchange, and effective publication and communication channels, and by generating cyberinfrastructures and platforms for interdisciplinary integration (Dietrich et al., 2014; Leonelli, 2013).

It is our contention, though, that the standardization of organisms, infrastructures and material conditions, in addition to the large social networks of scientific exchange that have been developed around model organisms, so aptly described by scholars like Ankeny and Leonelli, have in fact been centered on shaping model organisms as *model carriers* and not particular models per se.

Against the backdrop of what we have presented thus far, we can distinguish three different kinds of *X*-features present in model organisms (Fig. 4) that can be mobilized and interpreted in different scientific models in the way we discussed in the preceding sections: (1) the *evolved features* and traits that are the product of the evolutionary history of the selected organisms before being transported into laboratory settings; (2) the *standardized features* that are generated through standardization and preparatory experimentation; and (3) the *experimental features* that are introduced into *token* organisms under particular interventions and experiments. This distinction between the three types of *X*-features is prior to the participation of these features in models and representations. Thus, following the DEKI account, features of any type can be interpreted or non-interpreted, and exemplified or non-exemplified.

For example, *M. musculus* has a long tail, a hair coat with tactile and pelage hairs, erect, rounded ears, a pointed snout with vibrissae, poor thermoregulation, highly concentrated urine, and, compared to other mammals, a short generation time and large litters (evolved features). However, the inbred lab strains have been intentionally shaped to harbor considerably less phenotypic variation at different levels of organization (standardized features) than their conspecifics that have adapted to different urban and agrarian environments (Phifer-Rixey & Nachman, 2015). As a result of this, standardized mice perform different behaviors that organisms from natural populations do not exhibit (e.g., the male barbering behavior in cages). Additionally, different lines have particular ontogenetic, physiological, and morphological traits (experimental features) induced through manipulations (e.g., conditionally targeted mutations using site-specific Cre recombinases, injections of morpholinos and dsRNAi, and genomic editing through the CRISPR/Cas system).

To generate particular models, scientists jointly include some of the natural, evolved features of *M. musculus*, along with features that arose during the standardization phase, as well as experimental features that are the products of goal-driven manipulations. Importantly, the experimental features are always sought after within the purview of a given model (e.g., ectopic gene expression or gene silencing to probe the genetic causal basis of the morphogenesis of a particular structure shared by a given phylogenetic group). In contrast, the evolved and standardized features (sometimes also confusingly called the ‘wild type’ features of model organisms) are general and not model-specific.

## Modeling versatility continued

Having explicated the standardization of model organisms in the laboratory as involving the modification of their pool of carrier-features, we are now in a position to expound the last two types of modeling versatility that model organisms afford to scientists.

### Manipulation versatility

As Ankeny and Leonelli (2020) suggest, the epistemic access to phenomena and the representational capacity of model organisms are bestowed on them in part by the range of manipulations that can be performed on them to approximate or gain an understanding of the targets of different models. This *manipulation versatility* is the fifth dimension of modeling versatility that we consider accompanies model organism-based research, given the kind of model carriers it works with. For example, scientists can expand the genetic code of mice for the systematic in vivo study of cellular proteins (Han et al., 2017); they can generate either germ-free or microbiome-depleted organisms (see Kennedy et al., 2018) to investigate multifarious phenomena (e.g., sleep/wake cycles; Ogawa et al., 2020); or they can perform different kinds of gene targeting to render *M. musculus* specimens more suitable to peer into diseases of biomedical interest (Gurumurthy & Lloyd, 2019). Another example of manipulation-induced traits comes from so-called ‘humanized mice,’ which are immunodeficient mice that have been engrafted with primary hematopoietic cells or tissues from humans, giving rise to a functional ‘human’ immune system with the goal of studying relevant pathologies (e.g., conducting in vivo preclinical studies; Allen et al., 2019).

The versatile manipulation of *X*-features and properties of model carriers is, of course, not an idiosyncratic particularity of model organism-based research. After all, other material scientific models, say, machines, can also be standardized and amply manipulated through experimentation and accordingly acquire new *X*-features that can be interpreted, thus exemplifying particular features that can be then imputed to target systems by following local keys. This notwithstanding, we think that it is *the combination and extent of all the dimensions of modeling versatility* expounded herein, and not any single dimension on its own, what makes model organism-based research unique in the scientific landscape.

### Discovery versatility

It is important to stress that the standardization process and the preparatory experimentation on model organisms do not eliminate or modify all their evolved features—they only tinker with a subset of these—and there are many features that are not even known or expected by scientists. As biological research advances through the collective efforts of committed epistemic communities, more and more traits are discovered in model organisms, which increases the number of *X*-features that can be interpreted in different theoretical domains and mobilized within particular models with variable *T*- and *S*-scope. This unbolts new research directions when working with model organisms, for new knowledge (e.g., the discovery of the relevance of the microbiota–gut–brain axis for glial functions in mammals) can reconfigure our understanding of the target or which features we consider causally important. The possibility of discovering unexpected connections means the carrier organism is never fully neutral or passive. Instead, the model organism *qua* integrated system serves as a constraint on modeling choices, with its relevance unfolding over time.^9^ For this reason, we believe that there is an inescapable element of *serendipity* and *surprise* in model organism-based research: scientists employ model carriers that, while standardized to some extent, also contain many features that are mysterious and unfamiliar to them (see also Larraín, 2024). In this sense, model organisms are indeed different from the majority of material model carriers in the sciences, whose properties are *usually* well-known from the beginning—indeed, it is precisely because these properties are known that it is possible to assemble and generate these material carriers, say, compound machines or ball-and-stick models of molecules, in the first place.^10^

This is the sixth dimension of modeling versatility that we argue comes with model organisms as model carriers: what we call *discovery versatility.* This refers to the existence of unknown *X*-properties due to evolutionary history that, once revealed, can form part of modeling repertoires. For example, the discovery of the conservation of the neurotransmitter system in the brain of the zebrafish *Danio rerio*—which exhibits several monoaminergic localities with neuronal clusters for different neurotransmitters, similar to mammalian nervous systems—has allowed for certain neurodegenerative disorders to become novel targets of new models derived through research with this model carrier (Chia et al., 2022). In cases like these, inferences based on shared ancestry and evolutionary conservation come to bear as fundamental aspects for model organism-based research (as discussed by Levy & Currie, 2015).

This last dimension of the modeling versatility of model organism-based research has already been hinted at by Ankeny and Leonelli (2020, pp. 15, 17):

Of course, not all of the important biological characteristics of these organisms were evident when they were first obtained in the field (in their truly ‘wild’ form) […]. Experimental organisms have been engineered and modified to enable the controlled investigation of specific phenomena, yet at the same time they remain largely mysterious products of millennia of evolution, whose behaviours, structures, and physiology are often still relatively ill-understood by scientists. Through this hybrid status as both natural and artificial objects, experimental organisms facilitate exploratory research by enabling biologists to ask questions without necessarily having clear expectations about what answer they will obtain or even about what questions will end up being the focus of inquiry.

Their valuable points notwithstanding, we disagree with their general assessment on this issue: “The dual status of model organisms—at once samples of nature and human artefacts, simultaneously modeling known and unknown phenomena—is the feature that makes them such interesting objects in biological research, and indeed an important and distinctive type of scientific model” (Ankeny & Leonelli, 2020, p. 17). We do not think that model organisms as wholes model “unknown phenomena.” Rather, once hitherto unknown traits (*X*-features) are uncovered in the organismal model carriers, new models can be built that take these features into account. This may alter the available explicit targets (*T*-scope) in manifold organisms (*S*-scope) for model-building, opening up new representational functions for the model organism in question. Discovery versatility is partly explained by common facts that account for the other dimensions we have covered in this article: organisms are developmental systems with traits that change as a function of time and that are the result of complex and variegated evolutionary histories (with varying degrees of trait evolutionary conservation).

We must stress that our assertion of the discovery versatility afforded by organismal model carriers is not categorical; as we said above, we do not rule out that there are properties unknown to scientists in material carriers in other scientific disciplines that could then be capitalized upon to refine or build new models. We simply judge that, while this is rather infrequent in machines and static physical structures, it is *de facto* the norm with organisms: no species that has been brought into the laboratory is fully known beforehand. Conducting research with model organisms is precisely what helps scientists to unveil the *X-*features that may have representational implications. Therefore, the repertoire of models that can be generated with a specific model carrier can increase over time: the number of models derived from model organisms, tackling diverse targets and with varying scopes in both synchronic and diachronic terms, usually mounts as another outcome of the modeling versatility endowed by organismal carriers. This last point, of course, is merely a numerical one and does not imply that new models that accrue over older ones are necessarily or increasingly better (for discussions on the many limitations of model organism-based research, including its overly standardized approach, see, e.g., Bolker, 2012; Farris, 2020; Kunze & Malfatti, 2025).

Let us wrap up now to see what we have learned about the modeling potentialities of model organisms and how they differ from the modeling potentialities of other experimental organisms in the biological and biomedical sciences.

## From representational power to modeling versatility

An important tenet or assumption in model organism-based research is that model organisms exhibit a broader and more encompassing ‘representational scope’ than the one attributed to other experimental organisms. In fact, Ankeny and Leonelli (2020) suggest that they may be positioned at one end of the spectrum, exemplifying a remarkable level of generalizability that is rationalized, say, to justify biomedical research in organisms that do not belong to the phylogenetic vicinity of *H. sapiens* (for discussion, see also Green, 2024). As examples of this, we can mention how diabetes and insulin resistance diseases are investigated in *D. melanogaster* or how scientists establish yeast and filamentous fungi as model organisms for studying peroxisome biogenesis disorders (Graham & Pick, 2017; see van der Klei & Veenhuis, 2006). However, Ankeny and Leonelli (2020) attribute even more epistemic relevance to the ‘representational target,’ pointing out that manifold phenomena can be feasibly approached through experimentation on a model organism: from genetic and cell signaling mechanisms, physiological processes and developmental sequences, to ecological interactions and certain kinds of medium-range evolutionary dynamics.

Ultimately, it is the conjunction of both wide representational scope and wide representational target that confers model organisms their distinctive *representational power* in the biological sciences, according to Ankeny and Leonelli (2020). In their words, “what defines model organisms as a specific subclass of experimental organisms is the representational power attributed to them,” which is “grounded in the specific modes of intervention and standardisation used to establish and develop these organisms” (p. 9), and “stems from the simultaneous attribution of wide representational scope and wide representational target” (p. 10).

In sum, Ankeny and Leonelli make three interrelated claims regarding the representational properties of model organisms: (i) model organisms can be attributed representational target, scope, and power; (ii) representational power is the result of the combination of representational target and scope; and (iii) having high representational power (or, equivalently, simultaneously having broad representational target and broad representational scope) is what distinguishes model organisms from other experimental organisms in the scientific landscape. In what follows, we shall put into question these claims and offer an alternative position that captures Ankeny and Leonelli’s valuable core idea while avoiding inconsistencies.

Regarding claim (i), namely, that model organisms can be attributed representational target, scope, and power, we should begin by noticing that what Ankeny and Leonelli call ‘representational target’ in this context is what we called ‘total *T*-scope,’ namely, the set of all the target phenomena that are represented using a given model organism at every ontogenetic stage. Looked at this way, nothing prevents us from attributing ‘representational target’ to a model organism as long as it is clear that it is not the organism that has targets but the diverse models it supports as a carrier. In other words, ‘representational target’ does *not* capture the idea that a model organism has multiple targets—it doesn’t have targets because it is not a model—but that it supports *multiple models* built by scientists that engage in manifold epistemic activities (e.g., abstracting, theorizing, intervening) across disciplines, which represent different targets.

Similarly, Ankeny and Leonelli’s ‘representational scope’ is what we called ‘total *S*-scope,’ that is, the set of all the organisms or kinds of organisms in which the targets represented by all the models built from a given model organism occur. As anticipated in Sect. 2, the *S*-scope is a property of *models* and thus the problem with attributing ‘representational scope’ (i.e., total *S*-scope) to a model organism is that it masks the fact that a model organism can support models with broad and narrow *S*-scopes simultaneously or at different moments in its ontogeny (e.g., think of models of somitogenesis or gastrulation in *M. musculus* and their varying phylogenetic projectability; see also other examples in Sect. 4).

Finally, the fact that model organisms are not models with representational functions in themselves (for in-depth discussion, see Prieto & Fábregas-Tejeda, 2025) casts doubt over whether they can be attributed ‘representational power,’ which is a property of models (or representations more generally). Attributing representational power to a model organism blurs the fact that the model organisms can support different models with varying representational power. What represents in model organism-based research—and thus what has representational power—is not the model organism on its own but the models constructed from abstracted and interpreted parts of the model organism.

Apropos (ii), the idea that representational power is the result of the combination of representational target and representational scope (*sensu* Ankeny and Leonelli) seems unjustified. Indeed, it is not difficult to imagine scenarios in which a model has a narrower representational target and scope than another model but is more powerful nonetheless (e.g., because it allows for more accurate predictions, detailed and robust explanations, or possibilities of intervention, etc.). This is the case of many models built from non-organismal carriers in the biological sciences (e.g., organoids, cell cultures, etc.) that simultaneously have, in Ankeny and Leonelli’s terms, narrower representational target and scope *and* higher representational power than their model organism-derived counterparts in certain modeling contexts. For instance, a human organoid-derived model that represents a specific pathological phenomenon (narrow representational target) in humans (narrow representational scope) might be more powerful than, say, a mouse-derived model, due to its closer relatedness to the target system (see Kim et al., 2020).

This is connected to Ankeny and Leonelli’s claim (iii), which states that the difference between model organisms and other experimental organisms is that the former have higher representational power (due to their broader representational target and scope). We contend that, *from the point of view of scientific representation*, there is no substantial difference between model organisms, other “model systems” (e.g., organoids), and experimental organisms: they are all material systems playing the role of carriers that support the construction of models that represent specific phenomena. Representational power is a property of each of the models, and its degree does not depend on the type of carrier from which the models are generated.^11^ Consequently, the degree of representational power of this or that model cannot be used to demarcate model organisms from other types of experimental organisms and model systems.

In spite of our dissent with Ankeny and Leonelli’s claims, we do not deny the peculiar status of model organisms *vis-à-vis* other model systems and experimental organisms in the life sciences. We only argue that what is special about model organisms is not to be found in the notion of ‘representational power.’ Instead, we suggest that what characterizes model organisms is their relatively high *modeling versatility*^12^ in its several dimensions outlined in this paper, which are grounded on the developmental and evolved features of model organisms *qua* organisms, the process of standardization they undergo, and the integrative and complex modeling ecosystems in which they are embedded.

Of course, the demarcation between model organisms and other experimental organisms is a matter of degree rather than a qualitative one.^13^ When used in representational contexts, other experimental organisms might display the six dimensions of modeling versatility we have spelled out, albeit not at once and to a lesser extent than full-blown model organisms. Similarly, we see the demarcation between model organisms and other material carriers in the sciences as a matter of degree.^14^ Other carriers (e.g., the Phillips-Newlyn machine mentioned in Sect. 6.2, footnote 7) might afford some or even all dimensions of modeling versatility, but arguably not to the extent to which model organisms exhibit them. In sum, we submit that model organisms stand out in the biological and biomedical landscape because they display a set of dimensions of modeling versatility that likely no other material carrier in the sciences simultaneously and to the same extent has.

## Conclusions

In this paper, we have systematically investigated the second element in the compound expression ‘model *organism*’ to spell out the role these creatures play (i.e., as model carriers) in the representational practices of the life and biomedical sciences. We have shown why it is important to underscore that model organisms are material, integrated systems with an evolutionary history and an organization that changes over the course of their ontogeny, which also exhibit some traits that are generated through standardization and preparatory experimentation, for understanding the epistemology of model organism-based research.

In particular, we argued that what makes model organisms special is that, throughout their ontogenies, they possess features and properties that scientists marshal to provide six kinds of modeling versatility: (i) *synchronic target versatility*: at a particular ontogenetic moment of an organism, different models for investigating different target phenomena (*T*-scope) can be advanced; (ii) *synchronic scope versatility*: the *S*-scope of the models generated at any ontogenetic moment can vary and thus (whole) model organisms per se cannot be assigned any *de facto* representational scope; (iii) *diachronic target versatility*: the *X*-properties of organisms radically change throughout their ontogeny, and, in each developmental stage, biologists can assemble different models for varied target phenomena (i.e., the *T*-scope of the models that can be built with an organism varies throughout ontogenetic time); (iv) *diachronic scope versatility*: the changes that model organisms undergo throughout their ontogenies also impact the *S*-scope of the models than can be built by theoretically interpreting selected parts of the model organism; (v) *manipulation versatility*: biologists standardize and experimentally tinker with the *X*-properties of model organisms in their epistemic pursuits, and this yields an even wider array of ontogenetically possible models; and (vi) *discovery versatilit*y: model organisms have many unknown *X*-properties due to their diverging evolutionary histories, which, after being uncovered, can be theoretically interpreted and mobilized to build even more models.

We contended that model organisms are indeed special when compared with other model systems and experimental organisms in the life and biomedical sciences. Our original position on the issue, however, is that what is unique about model organisms is not a qualitative feature or something found in the misattributed notion of ‘representational power,’ as has been hitherto defended in philosophical scholarship. Instead, we suggest that what characterizes model organisms is a difference of degree: their relatively *high modeling versatility*—something that likely no other material carrier in the sciences (not least other experimental organisms) simultaneously possesses to the same extent in the six dimensions articulated herein.

Our contribution aimed to provide a generalized account of modeling versatility in model organisms, which comes at the expense of some detail and nuance. First, the dimensions of modeling versatility are conceptually distinct, and this is why we treated them separately. However, they are not mutually exclusive but interact in complex ways. For instance, discovery versatility grounds the unveiling of new features in model organisms, which opens up the possibility of modeling new target phenomena (synchronic and diachronic target versatilities) in other organisms (synchronic and diachronic scope versatilities).

Second, we acknowledge that different modeling and disciplinary contexts might shape the relevance and applicability of the six dimensions of versatility we have described. For instance, our claims about synchronic target and scope versatility may apply especially well in domains such as regeneration biology, where phenomena are bounded by clear outcomes and where stable background assumptions enable researchers to isolate parts of the organism for modeling. In contrast, these dimensions of modeling versatility might be less prominent in fields like behavioral neuroscience, in which maintaining a model that is open-ended and underdetermined rather than committing to a fixed “ground truth” too early can be epistemically advantageous due to the plasticity of behavior. In such cases, emphasizing versatility as the capacity to generate multiple tightly scoped models may be less useful than focusing on epistemic flexibility and ambiguity. Conversely, diachronic scope versatility may be especially relevant in behavioral studies, where patterns of behavior change across developmental stages, social contexts, or environmental exposures. This dimension, however, may be less applicable in domains that model fixed mechanisms, such as well-defined genetic or biochemical pathways. In these contexts, whether or not the organism changes over ontogeny is often irrelevant if the modeled mechanism is presumed to be stable or binary (i.e., present or absent).^15^

Third, our generalized account might convey an overly static picture of modeling processes with model organisms. However, we fully recognize that modeling is a dynamic activity and that the processes of selection, abstraction, and interpretation of elements within the carrier are not fixed from the outset. Researchers cannot always assume access to clear empirical knowledge about the full life cycle or relevant developmental windows of the organism under study. Rather, that knowledge must be acquired through the modeling process itself (for a more detailed account of the dynamic, exploratory nature of research with model organisms, see Larraín, 2024). In this sense, our account readily allows for the possibility that a previously irrelevant part may become important, or that a prior abstraction was mistaken, since it suggests that surprise challenges the boundary between the “relevant” and the “irrelevant,” as well as between interpreted and non-interpreted features.^16^

In scientific practice, biologists marshal different dimensions of modeling versatility both for generating well-established, stabilized lines of research and for fostering more plastic, open-ended exploratory investigations. Our framework is valuable because it is able to showcase that model organism-based research can have different orientations that capitalize on different combinations of modeling versatility (e.g., exploratory work hinges on discovery versatility in both synchronic and diachronic frames, and stabilized lines of research make use of standardized methods that yield manipulation versatility, usually in fine-grained ontogenetic snapshots).^17^

The fact that model organisms are not ‘theoretical models’ in a standard philosophical sense does not detract from their tremendous epistemic and representational importance in the life and biomedical sciences. On the contrary, revealing them as versatile model carriers shows their capacity for generating *countless models* of manifold targets with a wide phylogenetic spectrum of applicability—from the very broad to the very narrow. This versatility is the result of the conjunction between the materiality that organisms bring to the fore *qua* organized, developing living systems with an evolutionary history, and the processes of standardization and intervention they undergo in the laboratory.

## Footnotes

1 In the biological sciences, experimental organisms are sometimes called “Krogh organisms” (Green et al., 2018), “specialized models” (Liberali & Schier, 2024), or “satellite models” (Simpson, 2002) to indicate that, although they are like model organisms in many respects, they are used by particular research communities to investigate narrower sets of phenomena, sometimes in combination with research on model organisms. To emphasize these distinctions, model organisms proper are frequently qualified as “traditional,” “classic,” “established,” “standard,” “canonical,” “supermodels,” etc. In this paper, we speak of ‘model organisms’ and, for the sake of simplicity, refer to non-model organisms (including Krogh organisms) as ‘experimental organisms.’

2 The DEKI account of scientific representation is a philosophical framework developed by Roman Frigg and James Nguyen (2016, 2018, 2020, Chap. 8; Nguyen & Frigg, 2022, Chap. 4) and refined by Guido I. Prieto and Alejandro Fábregas-Tejeda (2025) to explain how scientific models represent aspects of the world. According to this account, a model consists of properties of a material or conceptual object—the so-called carrier—that are interpreted in terms of a certain theoretical domain. The model does not need to have a real target, but when it does, it represents it by virtue of denoting it, exemplifying some of its features, and imputing these features onto the target, customarily after they have been transformed using a key. These operations—denotation, exemplification, keying-up, and imputation—give DEKI its name. To give a simple example, in a balls-and-sticks model of molecular structure, the carrier is the material object made of balls and sticks. Some of the features of the carrier can be given a theoretical interpretation, say, the balls can be interpreted as atoms of different elements, the sticks can be thought of as chemical bonds between atoms, and so on. These features and their interpretation constitute a model of molecular structure, which might or might not represent a real target molecule in nature. When it is used to represent a concrete target, the model denotes (refers to) the molecule, and exemplifies some of the molecule’s properties (e.g., its spatial configuration). These properties can be translated into molecule-features via a key (e.g., the physical distances between balls in the carrier are converted into molecular distances) and then imputed onto the target molecule. When these operations take place, the model is said to be a representation of the target.

3 We employ the DEKI account because it provides a robust framework with which to assess the epistemology of model organism-based research (Prieto & Fábregas-Tejeda, 2025) and because we want to engage in a discussion with recent positions that use it as a general framework to assess representational practices with model organisms (Ankeny & Leonelli, 2020; Sartori, 2023). However, we do not hold a strong commitment to the DEKI account, and this particular view of models and scientific representation is not crucial for the main arguments advanced herein. As Frigg and Nguyen (2020) note, every view of models must include a theory about how carriers relate to models. Therefore, if model organisms are indeed carriers, views other than DEKI (e.g., inferentialism) might also account for the dimensions of modeling versatility that we will describe in what follows. Should a stronger contender emerge that provides greater epistemic insights for understanding modeling practices that employ model organisms, we would gladly adopt it. We are grateful to an anonymous reviewer who pushed us to think more carefully about this issue.

4 Although our focus here is on model organisms, the discussion that follows on representational target and representational scope is, in principle, applicable to scientific models in general.

5 Some biological practices require even more fine-grained temporal snapshots than developmental stages (for discussion, see Fábregas-Tejeda, 2025), so the synchronous construction of models (with corresponding *T*- and *S*-scopes) also refers to these finer temporal units. In this paper we mostly refer to developmental stages for the sake of expository simplicity.

6 We acknowledge that this particular point about material carriers enabling several models is not restricted to model organisms and is widely recognized in contemporary philosophy of science. Rather than claiming that the notion of synchronic scope versatility is completely novel, we argue that we are doing five related things that constitute our contribution: we are (i) making it explicit, (ii) grounding it in a concrete representational theory (i.e., our refined version of the DEKI account), (iii) illustrating it with numerous examples, (iv) giving it a name, and (v) inserting it into a taxonomy alongside other types of modeling versatility. For these reasons, we do not see our contribution on this point as merely an accommodation of extant positions in the philosophy of scientific modeling. We thank an anonymous reviewer for pushing us to clarify the degree of novelty of a notion such as “synchronic scope versatility.”

7 As we mentioned in footnote 5, we use developmental stages in this paper for convenience but recognize that there are cases where important target phenomena subject to scientific modeling are temporally more fine-grained than particular developmental stages (e.g., phenomena in early-onset gastrulation versus phenomena restricted to later time points in gastrulating embryos). Our claim for diachronically extended model construction with model organisms also covers these instances, but we will not foreground them in what follows.

8 Somites are blocks of tissue that later differentiate into, among other structures, vertebrae, the rib cage, skeletal muscle, cartilage, and tendons, depending on their location along the anterior-posterior axis.

9 We thank an anonymous reviewer for their suggestions that enriched our discussion on this point.

10 Exceptions exist, of course. A famous one in the scientific modeling literature was pointed out by Frigg and Nguyen (2020, p. 171; citing Morgan & Boumans, 2004, fn. 14). A leak in the Phillips-Newlyn machine—a hydraulic analog computer used to physically model economic processes by simulating monetary flows with water—turned out to be interpretable as money leaving the standard economy and entering a black market. This is a case whereby unexpected *X*-features in a machine model carrier open new lines for inquiry. We thank an anonymous reviewer for reminding us of this example.

11 Somewhat similar skepticism about the possibility of attributing representational power to model organisms has also been alluded to by Sartori (2023, p. 11). He suggests that representational target and scope—and, hence, representational power—are attributes of the specific *models* in which model organisms participate and thus “it does not seem to be the case, as Ankeny and Leonelli hold, that [model organisms] generally exhibit greater representational power than other experimental organisms.”

12 Our conclusion that modeling versatility rather than representational power is the hallmark of model organisms is compatible with a recent contribution by Juan Larraín. He shows that representational power does not play an important role in the selection, uses, and roles of model organisms, and argues that “the success of model organisms is based on their *flexibility* when performing exploratory research […] because this *plasticity* fosters the exploration of new possibilities and the accommodation of new perspectives” (Larraín, 2024, p. 19; emphasis added).

13 That there is no sharp line demarcating model organisms from other experimental organisms is apparent from the proliferation of terms that denote intermediate types of organisms between these two extremes. For instance, some model organisms are labeled as “non-canonical” (Holtze et al., 2021), “emerging” (Goldstein & Srivastava, 2022), or “non-model” (Russell et al., 2017) because they are lesser known than, or lack some features typically associated to, model organisms, such as standardization, resources, tools, and infrastructures.

14 We are grateful to an anonymous reviewer for pushing us to be more nuanced about the continuum between model organisms and other material carriers employed in scientific activities.

15 We thank an anonymous reviewer for their suggestions and for pushing us to be much more nuanced about the applicability of our account.

16 We thank an anonymous reviewer for raising this issue and prompting us to reflect on it more deeply.

17 We thank an anonymous reviewer for prompting us to make explicit this upshot of our work.
